# Influence of a Lubricating Gel (Orthospeed®) on Pain and Oral Health-Related Quality of Life in Orthodontic Patients during Initial Therapy with Conventional and Low-Friction Brackets: A Prospective Randomized Clinical Trial

**DOI:** 10.3390/jcm9051474

**Published:** 2020-05-14

**Authors:** Adrian Curto, Alberto Albaladejo, Javier Montero, Alfonso Alvarado

**Affiliations:** 1 DDS, Professor in Pediatric Dentistry, Faculty of Medicine, University of Salamanca, Avenida Alfonso X el Sabio s/n, 37007 Salamanca, Spain; 2 DDS, Professor in Orthodontics, Faculty of Medicine, University of Salamanca, Avenida Alfonso X el Sabio s/n, 37007 Salamanca, Spain; albertoalbaladejo@hotmail.com (A.A.); alfonsoalvaradolorenzo@gmail.com (A.A.); 3 DDS, Professor in Prosthodontics, Faculty of Medicine, University of Salamanca, Avenida Alfonso X el Sabio s/n, 37007 Salamanca, Spain; javimont@usal.es

**Keywords:** orthodontics, pain, oral health-related quality of life, oral health

## Abstract

The aim of this study was to investigate whether statistically significant differences exist regarding pain and the impact on oral quality of life of orthodontic treatment. A conventional brackets system was compared with low-friction brackets. A total of 90 patients (male = 35, female = 55) were chosen for this randomized clinical trial. Pain was assessed at 4, 8, and 24 hours and 2, 3, 4, 5, 6, and 7 days after the start of treatment using the McGill Pain Questionnaire. Oral health-related quality of life (OHRQoL) was assessed using the Oral Health Impact Profile-14 (OHIP-14) questionnaire. Oral quality of life was assessed at one month, with patients with low-friction brackets describing lower levels of pain. The patients with conventional brackets indicated a worse impact on their quality of life compared to the group with low-friction brackets. Statistically significant differences were found between the groups, with maximum pain observed between the first 24 and 48 hours, and the values of minimum pain are reached after 7 days. The pain and impact on oral quality of life was statistically worse in patients with conventional brackets compared to patients with low-friction brackets. The type of bracket system used was therefore shown to influence patients’ perceptions of pain and impact on their OHRQoL.

## 1. Introduction

Orthodontics is a dental specialty that has rapidly developed over recent decades. It deals with the correction of dental malocclusions and maxillary bone alterations through the application of forces that produce tooth movement [1]. During this process, various chemical mediators are released and give rise to the perception of pain that patients describe during their treatment [1,2].

According to several studies, approximately 90% to 95% of patients receiving orthodontic treatment experience pain during the first weeks of treatment [3,4]. This pain reaches its maximum level in the first 24–48 hours, and then gradually decreases until reaching a minimum level after about seven days [4,5,6].

Pain is an undesirable consequence of this orthodontic treatment. The pain described by patients may appear spontaneously, due to the force applied to the teeth in order for them to move, or as a consequence of functional processes, such as chewing [7]. This pain has a negative effect on patients´ desire to undergo orthodontic treatment and on their subsequent willingness to adhere to instructions [8].

Given that orthodontic pain in orthodontic patients is due to the application of forces that generate pressure, ischemia, inflammation, and edema in the periodontal tissues, some clinicians use lubricants to reduce friction and thus the perceived pressure on teeth [1]. Orthospeed® (Cosmodent Laboratories, Cantabria, Spain) is a product that acts as a lubricant and reduces friction between the arch and the bracket, resulting in continuous friction of a smaller magnitude. The gel is made up of oleic-based ingredients, has no adverse side effects, is tasteless, and does not stain teeth enamel [9].

The perception of pain depends on a person’s individual characteristics, such as age, sex, threshold of individual pain, stress, cultural differences, the magnitude of the force that is applied during treatment, and the emotional state of the patient. At present, there are only a few published studies concerning the impact on the oral quality of life of patients undergoing orthodontic treatment. However, the reports that do exist suggest that orthodontic treatment, which has an important psychosocial component, requires measures to assess the oral quality of life [10,11,12].

The purpose of this study was to analyze whether there were statistically significant differences regarding the perception of pain in the first seven days of orthodontic treatment and the impact on oral quality of life during the first month of orthodontic treatment. In addition, a conventional brackets system was compared to low-friction brackets and low-friction brackets alongside the use of lubricating gel (Orthospeed®). The null hypothesis was that the prevalence and severity of pain and the impact on oral health-related quality of life would not differ among the groups of patients.

## 2. Experimental Section

### 2.1. Ethics Approval and Patients Consent

The research project of this randomized clinical trial was approved by the Bioethics Committee of the University of Salamanca (USAL_16/060). The study followed the guidelines established by the Helsinki Declaration for research involving human subjects, as well as the CONSORT guidelines. The participants were informed about the examination procedures and assured regarding confidentiality of the collected information. Only those who gave written consent were included in the research.

### 2.2. Sample Size Calculation

Sample size calculation was performed using the Raosoft online sample-size calculator (Raosoft Inc, Seattle, Wash). With a margin of error of 5% and a confidence level of 95%, the target sample size was determined to be 90 patients, including 10% dropout. Previously published studies were considered when calculating the sample size [5,6,11]. All of the patients included in the study were recruited from the Dental Clinic of the University of Salamanca during 2019 and were treated by the same specialist. 

### 2.3. Randomization and Participants

This randomized clinical trial consisted of a total of 90 participants and was designed following the CONSORT guidelines (Consolidated Standards of Reporting Trials). The sample was divided into three groups, with each group consisting of 30 patients. The first group (CON group) consisted of patients who were fitted with conventional fixed-type multibrackets (Victory Series®, 3M, USA), the second group (LF group) was composed of patients who were fitted with low-friction fixed multibrackets (Synergy®, Rocky Mountain Orthodontics), and the third group (LFO group) consisted of patients who were fitted with low-friction fixed multibrackets (Synergy®, Rocky Mountain Orthodontics) and used Orthospeed®. In the treatment protocol, all of the employed brackets had 0.022" slots. The initial treatment arc was 0.014" super-elastic nickel-titanium (Nitinol®, 3M, USA). Brackets were ligated with 0.12-inch elastomeric ligatures (G&H Orthodontics). The patients were examined after four weeks to ensure the brackets were intact. After bracket bonding, archwires were inserted and ligated to all teeth in both the maxillary and mandibular arches. The archwire was cut distal to the molar tube without cinching back. During the single-blind study, the allocation of the bracket type of brackets was concealed from the participants but not the clinician. 

The inclusion criteria were as follows:

- Permanente dentition and aged between 18 and 40 years old;
- No previous orthodontic treatment;
- No craniofacial anomalies;
- No missing teeth, with the exception of third molars;
- Dental bone discrepancy between −1.5 and −7.0 mm in both arches; and
- Skeletal Class I or II malocclusion (ANB 0° and 5°) [13].

The exclusion criteria were as follows:

- Patients requiring tooth extractions;
- Mild or high dental crowding (between −1.5 and −7.0 mm);
- Patients who are not in a period of dental eruption;
- Significant physical or mental impairment;
- Patients with orthodontic-surgical treatment;
- Chronic use of analgesic, antidepressants, and/or anticonvulsants medications;
- Patients with previous pain-related pathology or disease (headache, migraine, or myalgia, among others);
- Dental caries;
- Pregnancy; and
- Patients with auxiliary orthodontic appliances.

No bite planes, lingual arches, or intermaxillary elastics were placed and no dental extractions were undertaken during the study period. Similar oral hygiene and appliance maintenance instructions were given to the four groups. Patients who declined to participate in the study were excluded from the sample. Patients who presented with periodontal pathology (gingivitis) were treated before starting orthodontic treatment to ensure that all patients began their orthodontic treatment with good oral health.

### 2.4. Study Design

In total, 90 patients were enrolled to increase the power of the study and compensate for possible dropouts. These patients were randomly divided into three equal treatment groups by using a randomization program (http://www.randomizer.org/form.htm), namely, conventional fixed-type multibrackets (Victory Series®, 3M, St. Paul, MN, USA), low-friction fixed multibrackets (Synergy®, Rocky Mountain Orthodontics, Denver, CO, USA), and low-friction fixed multibrackets (Synergy®, Rocky Mountain Orthodontics, Denver, CO, USA) with Orthospeed® (Cosmodent Laboratories, Cantabria, Spain) use. Bracket bonding, archwire insertion, and orthodontic treatment were performed by one nonblinded experienced operator between June and December 2019. Crowding was calculated according to dental bone discrepancy.

After bracket placement, the patients were presented with an analog visual scale (VAS) according to the McGill Pain Questionnaire from 0 to 10, with the terms "absence of pain" and "maximum pain" as endpoints. This scale evaluated the pain perceived by the patient at 4 hours (T1), 8 hours (T2), 24 hours (T3), 2 days (T4), 3 days (T5), 4 days (T6), 5 days (T7), 6 days (T8), and 7 days (T9) after the start of the treatment. One month after treatment, the patients completed the Oral Health Impact Profile-14 questionnaire (OHIP-14) to measure the impact on oral quality of life. The OHIP-14 questionnaire used in this study was previously developed by Slade (1997) as a shorter version of the OHIP-49 questionnaire introduced by Locker and Miller (1994). The OHIP-14 questionnaire was used to measure oral health-related quality of life; the Spanish version of this questionnaire was validated previously [14].

In this questionnaire, the participants were asked how often they had experienced impact with regard to each of the 14 items, and the responses were set on a 5-point Likert scale (0 = never, 1 = hardly ever, 2 = occasionally, 3 = fairly often, 4 = very often). This instrument was conceptually divided into seven subscales (pain, functional limitation, psychological discomfort, physical disability, psychological disability, social disability, and handicap) based on Locker’s theoretical model of oral health [15].

Impact was measured by totaling up the number of items recorded as occasionally or more often, thereby obtaining a quantitative variable that reflected patient wellbeing. Each domain was composed of two items, thus the impact score in each domain ranged from 0 to 2.

### 2.5. Statistical Analysis

The sample distribution (*n*, %) of the categorical variables and the means and standard deviations (mean ± SD) of the quantitative variables were used to describe the relevant variables in the sample. Descriptive statistics (means, standard deviations, and percentages) were calculated using SPSS v-20 (SPSS Inc., Chicago, IL, USA). The differences between the groups were analyzed using the ANOVA test with post-hoc Bonferroni correction for the quantitative variables. Pearson correlation coefficients were calculated to assess the linear relationships between the quantitative variables, respectively. The level of significance was set at 0.05.

## 3. Results

### 3.1. Characteristics of the Participants

**Figure 1:**
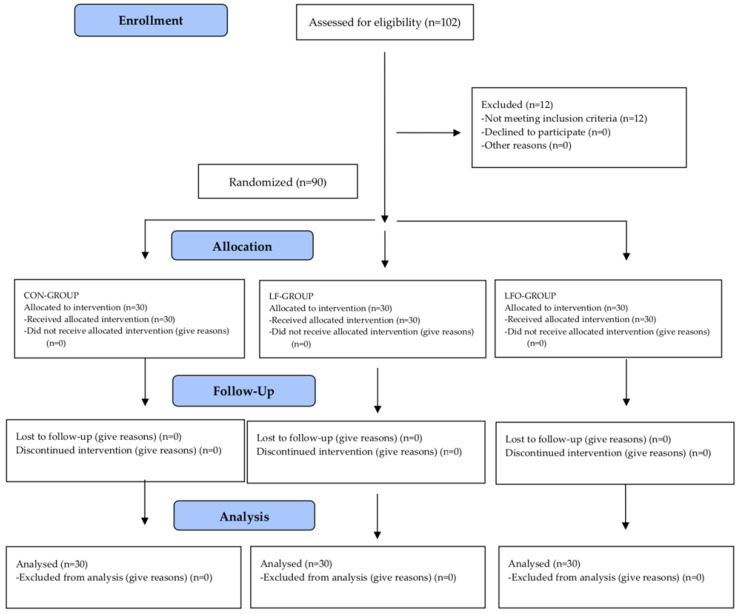
CONSORT flow diagram.

In total, 102 patients were screened from the Dental Clinic of the University of Salamanca during 2019. Twelve patients were excluded as they did not meet the inclusion and exclusion criteria, with a total of 90 patients meeting the screening criteria. All 90 patients consented and were randomly assigned to one of the three treatment groups by random tables, with equal numbers in each group (*n* = 30). A CONSORT diagram showing the flow of the patients through the study is given in Figure 1.

The mean age of the patients was 21.7 ± 9.9 years and was not significantly different between the three study groups. Of the 90 patients evaluated, 28.9% (35 patients) were men and 61.1% (55 patients) were female; this distribution was similar throughout all groups (Table 1). 

### 3.2. Pain Analysis

Statistically significant differences were found among the different groups throughout all assessment time points (*p* < 0.05). The patients with the conventional brackets exhibited had the highest mean value with respect to the level of pain during the entire first week after treatment (3.5 ± 2.4). In comparison, the group of patients with the low-friction brackets using Orthospeed® described the lowest level of pain on average (1.3 ± 1.9) for the same time period. This trend was observed throughout the entire follow-up period.

**Figure 2:**
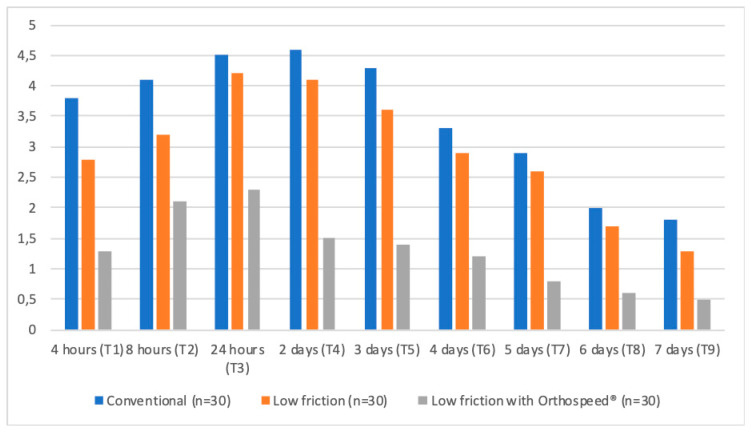
Pain among the different assessment time points using the visual analogue scale.

The peak of maximum pain, as indicated by the patients, was between the first 24 and 48 hours (Table 2). The patients with conventional brackets described their peak of maximum pain to be two days (4.6 ± 2.6) (*p* < 0.01) after the start of the study, with the patients with low-friction brackets, regardless of Orthospeed® use, indicating that their maximum level of pain occurred within the first 24 hours (2.3 ± 2.3 and 4.2 ± 2.7, respectively) (*p* < 0.01). From this peak, the level of pain gradually decreased until day 7 in all three groups (Table 2) (Figure 2).

In addition, a trend was observed after analyzing the influence of gender on the level of pain perceived during the first seven days, as greater levels of pain were perceived by women. However, this result was not statistically significant (Table 3), especially after six days in the group with low-friction brackets and in the first 4–8 hours in the group with low-friction brackets using Orthospeed®. Similarly, the Pearson correlation coefficient calculations showed that age did not significantly influence the level of pain perceived in the first seven days after treatment, although older patients generally perceived less pain (r = −0.1, *p* = 0.5).

### 3.3. Oral Health-Related Quality of Life Analysis

Table 4 compares the impact on oral quality of life among the treatment groups, showing that patients in the conventional brackets group reported the greatest impact on the different dimensions of oral quality of life, whereas the patients with low-friction brackets using Orthospeed® reported the least impact. Specifically, the pain score and overall impact averages of the conventional group (1.5 ± 0.7 and 3.0 ± 1.9) (*p* < 0.05) were significantly higher than the low friction group using Orthospeed® (0.9 ± 0.7 and 2.1 ± 1.4, respectively) (*p* < 0.05). As determined by the OHIP-14 questionnaire, pain, psychological discomfort, functional limitation, and physical disability were the most commonly affected dimensions. The impact on the handicap dimension was zero throughout all three study groups.

Regarding ofthe possible influence of gender on oral quality of life, Table 5 shows that women tended to perceive a greater impact on their oral quality of life compared to men. Moreover, statistically significant differences were found in the dimension of functional limitation in the group of patients with low-friction brackets. However, no significant influence of previous periodontal state was observed on the oral quality of life impact, but age was directly correlated with impact in the dimension of physical disability (r = 0.23, *p* = 0.03).

## 4. Discussion

This randomized clinical trial aimed to evaluate whether two bracket systems (conventional brackets versus low-friction brackets) influenced patient pain perception and oral quality of life, as well as the effect of the use of a lubricating gel (Orthospeed®) on the same descriptors. The limitations of the present study could be due to the short follow-up period in relation to the duration of the orthodontic treatment.

Published studies comparing low-friction Synergy® brackets with conventional brackets are based on in vitro investigations [16,17,18]. Some studies also evaluated the influence of lubricating gels on the degree of friction when comparing self-ligating and conventional brackets. These investigations were also in vitro and observed that the use of lubricating gels reduced friction between the arch and the bracket [19,20,21]. In agreement with the findings of this study, some published reports [22,23,24] concluded that patients undergoing orthodontic treatment described maximum pain peaks between the first 24 and 48 hours after treatment, and that the level of pain decreased from this point onwards until reaching minimum values after seven days. The visual analog scale is a useful tool to measure pain intensity and was used in most published orthodontic studies [24,25].

In this study, a significant discrepancy was observed between the pain caused by conventional brackets compared to low-friction brackets alongside the use of Orthospeed®. However, according to other authors, no statistically significant differences exist regarding pain between patients with conventional brackets and self-ligating brackets during the first week of treatment [24,26,27]. However, some studies with conflicting results concluded that lower levels of pain were experienced with the self-ligating bracket system [28]. The randomized clinical trial of Pringle et al. reported that patients with self-ligating brackets described a lower intensity of pain compared with patients fitted with conventional brackets [29].

In recent years, several questionnaires were developed to assess patients’ oral quality of life levels. In this regard, the OHIP questionnaire was the most frequently used in published studies and was also the questionnaire used in this work [30,31,32]. 

This study showed that the level of oral quality of life was significantly better at 30 days of treatment in the group using Orthospeed® compared to the group who were fitted with conventional brackets (Table 4). However, other studies focused on assessing the oral quality of life impact of patients with conventional brackets and self-ligating brackets concluded that there were no statistically significant differences, although the trend indicated the impact on oral quality of life is greater in the group fitted with conventional brackets.

Pain is the dimension that has the greatest impact on patients compared to the other dimensions of oral quality of life, as shown in Table 5. Our results also indicated that women report a greater negative impact than men, as previously pointed out by Mansor et al. [32]. The results of this study supported the findings of other studies, where it was concluded that orthodontic treatment has a negative impact on patients’ oral quality of life and causes pain to be experienced, especially during the first month of treatment. 

The primary limitation of this study was the sample size of 90 patients (30 patients per group). Of the 90 patients, 55 were female and 35 were male. In daily clinical practice, the frequency of female orthodontic patients is higher compared to male patients, as females are generally more concerned about their dental appearance than males. According to previous studies, gender does not influence the pain described by patients during orthodontic treatment [22,24,27,33,34]. In the future, a new randomized clinical trial with a larger sample size and equal gender distribution should be undertaken. The present study could help regarding this sample size calculation according to the calculated standard deviation values. 

To the best of our knowledge, this was the first randomized clinical trial reported in the literature, where the effect of a lubricating gel (Orthospeed®) and a conventional brackets system is compared to a low-friction bracket system to assess relative pain and the impact of orthodontic treatment on patients´ oral health-related quality of life. Future research could be directed to optimize the use of Orthospeed® in orthodontic treatment.

## 5. Conclusions

The peak of maximum pain was reached between the first 24 and 48 hours, at which time it started to decrease until reaching minimum values on the seventh day. Pain and the overall impact on oral quality of life was statistically worse in patients with conventional brackets compared to patients with low-friction brackets who used the lubricating gel (Orthospeed®).

## Figures and Tables

**Table 1:** Description and comparison of sociodemographic variables, age, sex, periodontal health, and dental bone discrepancy.

			Conventional (*n* = 30)	Low Friction (*n* = 30)	Low Friction with Orthospeed® (*n* = 30)	All (*n* = 90)
Age (Years)	Mean		23.5	18.87	22.6	21.7
	SD		12.6	3.6	9.5	9.9
Sex	Men	N	9	13	13	35
		%	30.0	43.3	43.3	28.9
	Women	N	21	17	17	55
		%	70.0	56.7	56.7	61.1
Periodontal Health	Healthy	N	18	25	24	67
		%	60.0	83.3	80.0	74.4
	Gingivitis	N	12	5	6	23
		%	40.0	16.7	20.0	25.6
Dental Bone Discrepancy	Upper	Mean	−2.4	−2.7	−3.1	−2.8
		SD	1.4	1.0	1.6	1.4
	Lower	Mean	−2.8	−2.3	−3.1	−2.7
		SD	1.4	0.9	1.5	1.3

**Table 2:** Comparison of pain levels throughout the different assessment time points using the visual analogue scale.

Time	Conventional (*n* = 30)		Low Friction (*n* = 30)		Low Friction with Orthospeed® (*n* = 30)	
	Mean	SD	Mean	SD	Mean	SD
4 hours (T1) **	3.8 ^a^	3.0	2.8 ^a,b^	3.1	1.3 ^b^	1.7
	ANOVA F: 6.467; gl: 2; *p* < 0.01					
8 hours (T2) *	4.1 ^a^	2.4	3.2 ^a,b^	3.1	2.1 ^b^	2.4
	ANOVA F: 6.4.184; gl: 2; *p* < 0.05					
24 hours (T3) **	4.5 ^a^	2.2	4.2 ^c^	2.7	2.3 ^b^	2.3
	ANOVA F: 7.333; gl: 2; *p* < 0.01					
2 days (T4) **	4.6 ^a^	2.6	4.1 ^c^	2.1	1.5 ^b^	1.8
	ANOVA F: 17.272; gl: 2; *p* < 0.01					
3 days (T5) **	4.3 ^a^	2.6	3.6 ^c^	2.1	1.4 ^b^	1.9
	ANOVA F: 13.640; gl: 2; *p* < 0.01					
4 days (T6) **	3.3 ^a^	2.5	2.9 ^c^	2.3	1.2 ^b^	1.9
	ANOVA F: 7.802; gl: 2; *p* < 0.01					
5 days (T7) **	2.9 ^a^	2.5	2.6 ^c^	2.3	0.8 ^b^	1.5
	ANOVA F: 9.065; gl: 2; *p* < 0.01					
6 days (T8) *	2.0 ^a^	2.2	1.7 ^a,b^	2.0	0.6 ^b^	1.7
	ANOVA F: 4.079; gl: 2; *p* < 0.05					
7 days (T9) *	1.8 ^a^	1.9	1.3 ^a,b^	1.8	0.5 ^b^	1.6
	ANOVA F: 3.656; gl: 2; *p* < 0.05					

* Statistically significant at *p* < 0.05. ** Statistically significant at *p* < 0.01. ^a,b,c^ The different superscript letters in the rows indicate which groups show significant differences according to the Bonferroni post hoc tests.

**Table 3:** Comparison of pain among the different assessment time points and according to gender using the visual analogue scale and the Student’s *t*-test.

Time		Conventional (*n* = 30)		Low Friction (*n* = 30)		Low Friction with Orthospeed® (*n* = 30)	
		Mean	SD	Mean	SD	Mean	SD
4 hours (T1)	M	3.3	2.8	1.1	1.3	1.9	2.8
	W	4.0	3.0	1.5	2.0	3.5	3.2
8 hours (T2)	M	3.8	1.9	1.9	2.7	2.2	3.2
	W	4.2	2.7	2.2	2.3	4.0	3.0
24 hours (T3)	M	4.4	1.9	2.0	2.0	3.7	2.4
	W	4.5	2.4	2.5	2.5	4.6	2.9
2 days (T4)	M	4.0	2.5	1.3	1.1	4.1	2.1
	W	4.9	2.6	1.7	2.2	4.2	2.2
3 days (T5)	M	3.9	2.8	1.3	1.3	3.3	1.8
	W	4.4	2.6	1.4	2.3	3.8	2.3
4 days (T6)	M	3.1	2.5	1.1	1.7	2.6	2.2
	W	3.4	2.6	1.2	2.0	3.2	2.5
5 days (T7)	M	2.8	2.5	0.5	0.8	2.3	1.8
	W	3.0	2.5	1.0	1.9	2.9	2.6
6 days (T8)	M	1.8	2.3	0.1	0.3	1.4	1.7
	W	2.1	2.2	1.1	2.2	2.0	2.2
7 days (T9)	M	1.4	1.6	0.2	0.6	1.1	1.6
	W	1.9	2.1	0.8	2.1	1.5	2.0

M: men; W: women.

**Table 4:** Comparison of the impact on the dimensions of oral quality of life.

Dimensions	Conventional (*n* = 30)		Low Friction (*n* = 30)		Low Friction with Orthospeed® (*n* = 30)	
	Mean	SD	Mean	SD	Mean	SD
Functional limitation	0.5	0.7	0.3	0.4	0.3	0.5
	ANOVA F: 1.502; gl: 2; *p* = 0.228					
Pain *	1.5 ^a^	0.7	1.1 ^a,b^	0.8	0.9 ^b^	0.7
	ANOVA F: 4.653; gl: 2; *p* < 0.05					
Psychological Discomfort	0.4	0.6	0.6	0.6	0.4	0.6
	ANOVA F: 0.482; gl: 2; *p* = 0.619					
Physical disability	0.4	0.6	0.4	0.7	0.3	0.5
	ANOVA F: 0.208; gl: 2; *p* = 0.812					
Psychological disability	0.1	0.3	0.1	0.4	0.1	0.2
	ANOVA F: 0.462; gl: 2; *p* = 0.631					
Social disability	0.1	0.3	0.1	0.2	0.1	0.4
	ANOVA F: 0.144; gl: 2; *p* = 0.866					
Handicap	0.0	0.0	0.0	0.0	0.0	0.0
Total OHIP *	3.0 ^a^	1.9	2.4 ^a,b^	1.6	2.1 ^b^	1.4
	ANOVA F: 2.784; gl: 2; *p* = 0.04					

* Statistically significant at *p* < 0.05. ^a,b^ The different superscript letters in the rows indicate which groups show significant differences according to the Bonferroni post hoc tests.

**Table 5:** Linear correlation between the sex of the individual and the impact on oral quality of life throughout the study groups according to the Oral Health Impact Profile (OHIP).

Dimensions		Conventional (*n* = 30)		Low Friction (*n* = 30)		Low Friction with Orthospeed® (*n* = 30)	
		Mean	SD	Mean	SD	Mean	SD
Functional limitation	M	0.7	0.9	0.1 *	0.3 *	0.5	0.5
	W	0.4	0.7	0.4 *	0.5 *	0.2	0.4
Pain *	M	1.3	0.7	1.0	1.0	0.7	0.8
	W	1.6	0.7	1.2	0.7	1.1	0.7
Psychological Discomfort	M	0.3	0.5	0.5	0.5	0.2	0.4
	W	0.5	0.7	0.6	0.6	0.6	0.7
Physical disability	M	0.3	0.5	0.2	0.4	0.4	0.7
	W	0.4	0.7	0.5	0.8	0.2	0.4
Psychological disability	M	0.0	0.0	0.2	0.6	0.0	0.0
	W	0.1	0.4	0.1	0.2	0.1	0.2
Social disability	M	0.0	0.0	0.0	0.0	0.0	0.0
	W	0.1	0.3	0.1	0.2	0.1	0.5
Handicap	M	0.0	0.0	0.0	0.0	0.0	0.0
	W	0.0	0.0	0.0	0.0	0.0	0.0
Total OHIP *	M	2.7	1.4	2.0	1.8	1.8	1.2
	W	3.1	2.1	2.8	1.4	2.3	1.6

* Statistically significant differences (*p* < 0.05) between men and women of the same group. M: men; W: women.
